# Is a Patient's Current Address of Record a Reasonable Measure of Neighborhood Deprivation Exposure? A Case for the Use of Point in Time Measures of Residence in Clinical Care

**DOI:** 10.1089/heq.2017.0005

**Published:** 2018-05-01

**Authors:** Andrew J. Knighton

**Affiliations:** Intermountain Institute for Healthcare Delivery Research, Intermountain Healthcare, Salt Lake City, Utah.

**Keywords:** geographic mobility, neighborhood deprivation, persistence, social determinants

## Abstract

**Purpose:** Interest is increasing in the use of geocoded patient address data to understand the effects that social determinants of health have on healthcare outcomes. Use of a patient's current address of record is often problematic given population mobility. Intragenerational economic mobility research suggests that patients will reside within neighborhoods with similar relative deprivation over time despite geographic mobility. The purpose of this study was to measure evidence of patient neighborhood deprivation persistence given a change in address of record.

**Methods:** A retrospective cohort study of patients receiving active care in an integrated delivery system in a high-mobility United States region. Neighborhood deprivation was measured using a block-group level area deprivation index. Neighborhood deprivation persistence was measured as the probability that an individual with an address of record change remained within a neighborhood with a similar deprivation score. Logistic regression was used to conduct multivariate analysis.

**Results:** Geographic mobility was highest among patients living in the most deprived neighborhoods versus least-deprived (odds ratio 1.75; 95% confidence interval: 1.71–1.79). Seventy-eight percent of all patients with a change of address did so to a neighborhood with a similar deprivation quintile. The probability that a random patient selected from the study had a change of address outside the same or neighboring quintile within a 1-year period ranged from 2% to 13%.

**Conclusions:** Neighborhood deprivation persistence was high among this population of patients from a high mobility region. A current address of record is a reasonable indicator of patient exposure to neighborhood deprivation within a 1–3-year timeframe that is useful in evaluating healthcare disparities.

## Introduction

As healthcare delivery organizations accept more responsibility for patient outcomes, interest in geocoded patient address data is increasing.^1–5^ This interest is being driven, in part, by a need to understand the effects that immediate and/or long-term exposure to one's neighborhood of residence has on healthcare outcomes. Increased neighborhood deprivation has been linked to variations in care and treatment outcomes, including delays in diagnosis and treatment,^6–8^ poor treatment adherence,^9,10^ and increased short-term mortality.^11–14^ It has also been associated with the disproportionate use of healthcare services, including higher emergency department (ED) and inpatient utilization, increased readmission risk, and overall increased per patient costs.^15–22^ The effect of neighborhood deprivation exposure on health is generally regarded as a longitudinal phenomenon best measured over an individual's life course.^23,24^ Increased exposure to the effects of neighborhood deprivation over time increases the risk of poor health outcomes.^25–28^

A patient's current address of record is a commonly used and readily accessible neighborhood identifier useful in quality improvement. However, a current address of record is potentially problematic in measuring patient neighborhood deprivation exposure. Intuitively, higher mobility rates in the population should increase the risk that a patient's current address of record recorded by the healthcare system is not the patient's actual current address of residence. This would seem increasingly true for patients receiving less frequent care although this has not been studied. Healthcare organization access to longitudinal patient address data is often difficult to obtain from source systems. These factors can impede the use of recent address of record data as reliable measure of actual neighborhood exposure given the risk of measurement error bias.

Stepping back, intragenerational economic mobility research in the United States and in other western countries would support the hypothesis that, despite geographic mobility, populations tend to reside within neighborhoods with similar relative socioeconomic strata over time.^29–31^ Spatial neighborhood deprivation persistence is also strong for ethnic minority groups.^28,32^ Neighborhood deprivation persistency then may mediate the effects of measurement error bias when using a patient's current address of record to measure patient neighborhood deprivation exposure.

The purpose of this preliminary study was to measure baseline patient address of record change rates overall and by neighborhood deprivation exposure in a population of integrated care delivery system patients in a state with higher than average 1-year mobility rates (17.6%) versus the national average (14.7%).^33^ Demographic risk factors associated with most recent address of record change rates were also measured. We then analyzed a subset of patients (for which geocoded data were available) who changed their address of record during the study period to evaluate persistence in observable neighborhood deprivation characteristics by the patient over time. If persistence is high after adjusting for known risk factors, then use of a recent single point-in-time address of record may be sufficient to identify patients exposed to neighborhood deprivation and its effects within certain timeframes. Reasonably accurate identification of exposed patients is useful in tailoring quality improvement interventions designed to mitigate the impact of neighborhood deprivation exposure on treatment outcomes.

## Methods

A retrospective observational study was conducted using a cohort of patients receiving care at Intermountain Healthcare (IH), an unaffiliated, nonprofit, integrated delivery system with 22 hospitals and 185 clinics serving patients in the Intermountain West. Given the lack of neighborhood deprivation information for patients outside Utah, this study was limited to active patients receiving care in Utah. Active patients (*n*=490,228) included those with at least one encounter from January 1, 2014 to August 31, 2015 (baseline measurement date) and a second encounter between August 31, 2015 and October 31, 2016 (study end point measurement date). These dates coincide with the timing of the roughly annual batch process currently deployed by IH to update geospatial patient information. An encounter includes an outpatient, ED or inpatient visit. Demographic data (including address data) were drawn from the organization's electronic data warehouse. Address data are drawn from the patient registration system. As a standard procedure, patient address information is reviewed and updated as needed at each clinical encounter. All address updates in the patient registration system occur as a result of this control process. Given this, to minimize the risk of false negative results (a patient noted as not moving when in fact they did), the study cohort was limited to active patients whose addresses would have been validated during both the baseline and follow-up clinical encounter. Minimal exclusions include patients who died during the study period, had a Post Office Box address as of the baseline address measurement date, and/or the geocoded information regarding their baseline address measurement date was unavailable. A demographic profile of the final study population (*n*=490,228) is noted in Table 1.

**Table 1.** **Cohort Demographics and Observed Probability of Change of Address by Patient Characteristic**

Patient characteristic	Category	Count	% Total	Observed probability of change of address	Risk factor for change of address (*p*<0.05)
*n*	Total	490,228	100.0	0.272	
Sex	Male	212,088	43.3	0.259	^*^
	Female	278,130	56.7	0.282	
Race	White	453,836	92.5	0.267	^*^
	Black/African American	5402	1.1	0.394	
	Asian	7112	1.5	0.260	
	American Indian	2770	0.6	0.328	
	Hawaiian	6337	1.3	0.353	
	Other	13,444	2.7	0.316	
	Unknown	1327	0.3	0.427	
Ethnicity	Hispanic	47,085	9.6	0.334	^*^
	Non-Hispanic	443,143	90.4	0.265	
Marital status	Married	237,439	48.4	0.231	^*^
	Single	196,935	40.2	0.310	
	Other	55,854	11.4	0.313	
Age	<18	117,531	24.0	0.287	^*^
	18–30	67,256	13.7	0.417	
	30–50	120,724	24.6	0.310	
	50–70	116,218	23.7	0.200	
	70+	68,499	14.0	0.160	
Area deprivation index quintile	1—least deprived	130,394	26.6	0.229	^*^
	2	132,016	26.9	0.241	
	3	91,520	18.7	0.282	
	4	72,687	14.8	0.309	
	5—most deprived	63,611	13.0	0.366	
Residence	Urban	474,456	96.8	0.273	^*^
	Rural	15,772	3.2	0.251	

^*^ Risk factor for change of address during study period (*p*<0.05).

Change in the address of record within the 14-month measurement window equates to a 33-month potential timeframe associated with an actual physical residence change. This is due to the lag between when a patient actually changes their physical residence and when they report this change at their next clinical encounter.

The primary outcome measure was the percentage of patients that changed their current address of record (estimated % change–address of record) overall and by key demographic factors, including neighborhood deprivation status. The current street address of record as of October 31, 2016 was compared to the baseline street address of record on August 31, 2015. Patients were identified as having a change of address of record if the study endpoint measurement date street address of record was different than the baseline measurement street address of record. Given data constraints, multiple addresses of record changes for each patient were not separately identified.

Neighborhood deprivation status was measured using an area deprivation index (ADI).^2^ An ADI is a geographic area-based measure of the disadvantaged position of residents relative to the society. The ADI developed for this study was calculated for the state using an ADI developed by Singh based upon 17 United States Census measures associated with mortality, including living conditions, income, unemployment, and education.^34^ A patient is assigned an ADI score based upon their address of residence. To simplify the interpretation of ADI information, ADI scores were grouped by quintile. Patients in the first or lowest quintile were those from the least deprived neighborhoods.

Potential risk factors associated with mobility were also analyzed for the population. These included sex, race (white, black/African American, Asian, American Indian, Hawaiian, Other, Unknown), ethnicity (Hispanic/not Hispanic), marital status (married/single/other), age (<18, 18–30, 30–50, 50–70, 70+), and residence (urban/rural). These characteristics were based upon patient self-reported data captured at the clinical encounter. Patient urban/rural classification was based upon the United States Census block group designation of urban, rural, or mixed given the address of residence. Areas with mixed designation were assigned urban/rural based upon where the majority of the population within the block group lived.

Measurement of deprivation persistence among patients with a change in their address of record was done using geocoded patient addresses as of August 31, 2015 and again as of October 31, 2016. Deprivation strata using ADI quintiles were measured for both the baseline and target address of record. Neighborhood deprivation persistence was measured as the percentage (%) probability that an individual with an address of record change remained within a neighborhood with a similar ADI quintile during the study period. A separate persistence measure was also developed looking at patients who changed address within the same or neighboring quintile (±1 quintile difference for those in the middle three quintiles, or +2 or −2 for those patients in the first or fifth quintiles, respectively).

Summary descriptive statistics were generated. Logistic regression was used to conduct univariate and multivariate analysis of risk factors associated with change of address of record rates overall and to measure neighborhood deprivation persistence over the study period. Necessary Institutional Review Board approval was obtained.

## Results

**FIG. 1.**
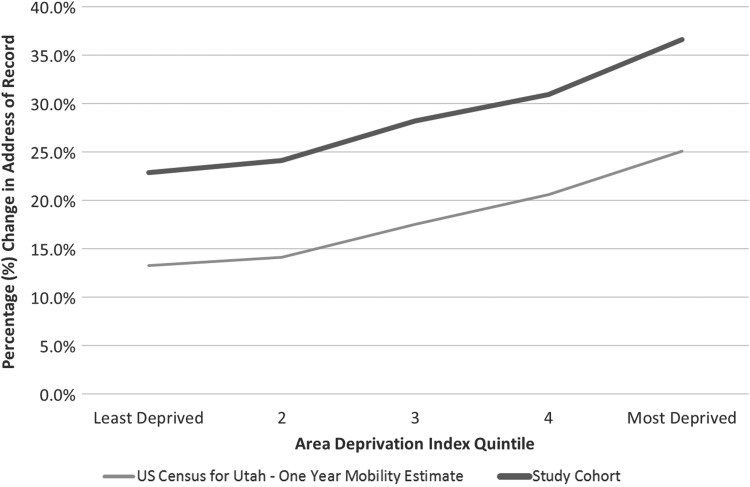
Observed percentage change in address of record in 1 year by ADI quintile, United States Census 1-year mobility estimate for Utah versus study cohort. ADI, area deprivation index.

The overall percentage of patients who changed their address of record during the 14-month study period was 27.2%. A 1-year pro-rata calculation of change of address of record activity for the same population was estimated at 23.3%. A comparison of the overall and 1-year IH pro-rata change of address rates with the United States Census 1-year mobility estimates matched by block group is included in Figure 1. The difference in the overall change of address rate was meaningfully different between the IH cohort and United States Census results for the state of Utah (17.6%), primarily due to limiting the study cohort to active patients only. Both measures showed a similar increase in change of address of record rates with an increase in ADI quintile (and deprivation). Increased neighborhood deprivation was associated with an increased likelihood of mobility during the study timeframe.

Demographic factors significantly associated with the odds of changing a patient's address of record during the study period included age, race, sex, ethnicity, marital status, residence location (urban vs. rural), and ADI quintile as noted in Table 1. Those most likely to change an address of record during the study period included young adults aged 18–30, some nonwhite minority groups, those of Hispanic ethnicity, individuals who were divorced or separated, patients living in urban block groups, or patients living in the most deprived neighborhoods.

**FIG. 2.**
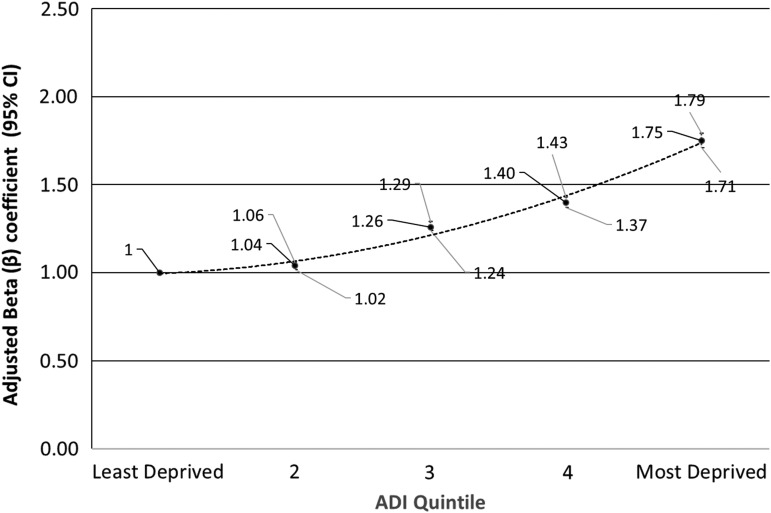
Adjusted odds of change in address of record during the study timeframe, by ADI quintile adjusting for age, sex, race, ethnicity, marital status, and residence.

Including adjustments for significant demographic factors noted in the previous paragraph, the most deprived patients living in the top area deprivation quintile were 1.75 times more likely to have a change of address of record in the past year (odds ratio [OR] 1.75; 95% confidence interval [CI]: 1.71–1.79) than patients in the first quintile as noted in Figure 2. A significant relationship between change of address rates and neighborhood deprivation remained evident after adjusting for known risk factors. Separating results by adults and children (<18 years of age), while children were generally less likely than adults to change their address of record, children in the highest quintile were 2.13 times more likely to change their address of record as pediatric patients in the first quintile (OR 2.13; 95% CI: 2.04–2.23).

**FIG. 3.**
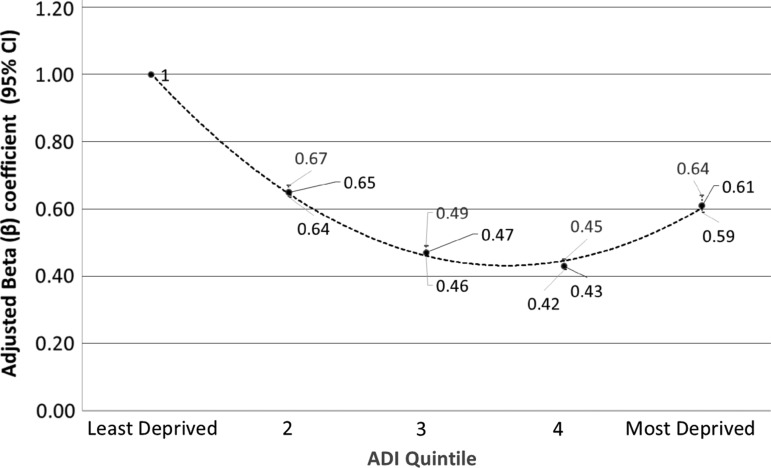
Adjusted odds of change in address of record to a different address of record within the same ADI quintile, by ADI quintile adjusting for age, sex, race, ethnicity, marital status, and residence.

Analyzing deprivation persistence over time, 42% of patients that changed addresses changed to a new residence in the same ADI quintile. Observed change of address of record percentages to the same quintile were highest for the least deprived neighborhoods (quintile 1=55%; quintile 2=44%) and most deprived neighborhoods (quintile 5=43%). Relaxing the measure of deprivation persistence, 78% of patients changed their address of record to either the same quintile or to a neighboring quintile as defined earlier. Similar results (not shown) were observed when separately analyzing adults 18 years of age and older and children (<18 years of age). Risk factors associated with the likelihood of a change of address of record within the same deprivation quintile included ethnicity, marital status, older age (>50), and rural residence. Adjusting for these risk factors, patients in the lowest deprivation quintile had the highest odds of remaining within the same quintile after moving, followed by patients in the second and fifth quintiles as noted in Figure 3.

Given these findings, the probability that a random patient included in the study had a change of address outside the same quintile within a 1-year period was 10%. The observed probability that a similar patient moved outside the same or neighboring quintile was 4%. These results varied based upon the baseline ADI quintile as noted in Table 2.

**Table 2.** **Given a Current Patient Address of Record, the Observed Probability of a Change in Address of Record Outside Same or Neighboring Area Deprivation Index Quintile, by Area Deprivation Index Quintile**

	Probability estimate by area deprivation index (ADI) quintile				
Deprivation persistence measure	Least deprived	2	3	4	Most deprived
One-year probability of change in address of record—estimate (A)	0.15	0.15	0.17	0.18	0.22
Probability of change outside same ADI quintile, given a change in address of record (B)	0.45	0.56	0.63	0.66	0.57
Probability of a change in address of record outside same ADI quintile (A×B)	0.07	0.09	0.11	0.12	0.13
Probability of change outside same or neighboring ADI quintile, given a change in address of record (D)	0.14	0.22	0.27	0.27	0.20
Probability of change in address of record outside same or neighboring quintile (A×D)	0.02	0.03	0.05	0.05	0.04

A neighboring quintile is defined as a change of address of record within the same or neighboring quintile ±1 quintile difference for those in the middle three quintiles, or +2 or −2 for those patients in the first or fifth quintiles, respectively.

ADI, area deprivation quintile.

## Discussion

Evidence of persistence in neighborhood deprivation exposure in patients following a change in address of record provides support for the use of recent point-in-time measures of patient social determinants to evaluate the short-to-intermediate-term effects of neighborhood deprivation in delivering clinical care.

Despite absolute differences in Utah mobility rates between the United States Census and the study cohort, the study demonstrates that patients from more deprived neighborhoods have higher mobility rates. Persons with characteristics associated with deprived neighborhoods were more likely to change their address.^2^ This may be due to housing, family, or employment-related factors that affect mobility. Housing-related factors include the need for cheaper housing or the desire for a better neighborhood with lower crime.^35^ Individuals were also more likely to move due to foreclosure or eviction.^35^ Family-related factors include the increased likelihood of establishing one's own household, common among younger adults leaving home for the first time.^35^ Employment-related factors tied to mobility include higher job-loss rates and a desire to be closer to work.^35^

Patients who changed their address of record maintained similar neighborhood deprivation exposure in their new neighborhood. Seventy-eight percent of patients who had a change of address remained within the same or a neighboring deprivation quintile in a roughly 1–3-year timeframe. We did not find meaningful differences in persistency rates between adult and pediatric populations. As noted earlier, work by Kunz et al.,^30^ for example, found a high degree of persistence in observable neighborhood characteristics in a sample of children despite geographic mobility over 5 years.^31^ Using neighborhood histories, van Ham et al., observed that individuals in Stockholm, Sweden, exposed to poverty concentration neighborhoods upon leaving home as adults are more likely than others to be exposed to poverty concentration neighborhoods over their life course.^31^ Spatial neighborhood deprivation persistence is also strong for ethnic minority groups.^28,32^ Economic mobility can be closely tied to geographic mobility over time.^36^ Other longitudinal studies in the United States have found that most economic mobility over time occurs over fairly small bands within the income distribution.^37–39^ Among adults, a study examining relative economic mobility rates over a 10-year period (1994–2004) for individuals 25–44 years old beginning in the bottom income quintile found that 55% remained within the same income quintile 10 years later relative to their peers. Twenty-five percent advanced one quintile. Downward relative mobility rates during this same timeframe were similar.^40^

Neighborhood deprivation persistence over time in adult and pediatric patient populations has implications in the use of patient geospatial data in quality improvement. The primary risk in using address of record data to measure social determinants is that a change of address occurs that is not recorded by the healthcare system. Our study found that the probability a patient's change of address of record outside their existing quintile ranged from 7% to 13%. The probability of an address change outside of the same or neighboring ADI quintile ranged from about 2% to 5%. Given these findings, the probability that a given patient had a change of address of record and that it led to meaningfully different neighborhood deprivation exposure versus what is currently recorded is relatively low. Evidence of persistence would suggest a recent address such as a patient's address of record is a reasonable point-in-time measure of patient exposure to neighborhood deprivation over a roughly 1–3-year window. Further longitudinal work is needed within delivery system research to understand neighborhood deprivation persistence over longer time frames; to measure a likely neighborhood deprivation exposure window based upon a patient's current address; and to evaluate the effects of immediate and long-term exposure to neighborhood deprivation on healthcare outcomes.

This study was retrospective in nature. As a result, no causal inferences can be developed from the results. Strengths of this study include a large sample size and use of a study population in a high-mobility region of the United States. However, the study was limited to patients who received care within an integrated care delivery system within a community with distinct characteristics. No physical validation of the address of residence was conducted. Using patient's address of record data to identify a change of address, there is a risk that a patient was identified as having changed the address of record without having physically moved locations. This may result from a minor edit or correction of the baseline address in the follow-up measurement period. To assess the potential risk, we compared the observed mobility rates overall and by ADI quintile against United States Census figures by block group. While change of address rates was lower in the IH test population, the differences were directionally consistent.

The more substantial risk to the findings is that a patient was identified as having not changed the address of record when in fact he/she had physically moved locations. This might result from the timing and frequency of actual patient encounters or from loss to follow-up. To limit the risk of a false negative result, the study cohort was limited to patients who had at least one encounter from January 1, 2014 to the baseline measurement point (August 31, 2015) and a second encounter during the follow-up study period. Measured change of address probabilities were higher overall in this subcohort, but relative findings by ADI quintile were consistent with United States Census results for Utah.

Regarding loss due to follow-up, patients included in the baseline period may no longer be active patients at the follow-up measurement point, given a change in insurance coverage and/or physically leaving the region. In addition to the items noted above, the risk of loss to follow-up is further minimized given the healthcare organizations scope of services in the region and the relatively low rates of mobility to locations outside the coverage area. However, we were unable to measure the percentage of patients that may have left the coverage area and the ADI score for their destination location.

Patients were excluded who did not have an address of record or the address was identified as a post office box. Patients excluded due to these factors were more likely to be male, non-Hispanic, divorced or separated, or older than the age of 50 years. Significant differences between the excluded patients and the final study population were due in large part to sample size. It may also reflect a distinct demographic group of patients who were underrepresented in the final results.

Naturally occurring groups such as families may have more similar observations than different groups resulting in correlated data. Data regarding family groupings were unavailable to eliminate the effects of correlated data on overall study results.

## Conclusion

Neighborhood deprivation persistence is high within this population of patients living in a higher than average mobility region. Given the measured level of deprivation persistence among patients, when coupled with other factors available to a clinician upon presentation, a recent address provides a reasonably accurate indicator of a patient's exposure to neighborhood deprivation within a short-to-intermediate-term timeframe. Evaluation of deprivation persistence over longer timeframes and in areas of the United States with differing characteristics is needed. Proper identification of at-risk patients living in more deprived neighborhoods can inform the design of tailored quality improvement solutions to improve healthcare outcomes in these more vulnerable populations.

## Abbreviations Used

ADI: area deprivation index
CI: confidence interval
OR: odds ratio
